# Analysis of a Multi-component Multi-stage Malaria Vaccine Candidate—Tackling the Cocktail Challenge

**DOI:** 10.1371/journal.pone.0131456

**Published:** 2015-07-06

**Authors:** Alexander Boes, Holger Spiegel, Nadja Voepel, Gueven Edgue, Veronique Beiss, Stephanie Kapelski, Rolf Fendel, Matthias Scheuermayer, Gabriele Pradel, Judith M. Bolscher, Marije C. Behet, Koen J. Dechering, Cornelus C. Hermsen, Robert W. Sauerwein, Stefan Schillberg, Andreas Reimann, Rainer Fischer

**Affiliations:** 1 Fraunhofer Institute for Molecular Biology and Applied Ecology (IME), Aachen, Germany; 2 RWTH Aachen University, Institute of Molecular Biotechnology, Aachen, Germany; 3 Zentrum für Infektionsforschung, Wuerzburg, Germany; 4 TropIQ Health Sciences, Nijmegen, The Netherlands; 5 Radboud university medical center, Nijmegen, The Netherlands; Universidade Federal de Minas Gerais, BRAZIL

## Abstract

Combining key antigens from the different stages of the *P*. *falciparum* life cycle in the context of a multi-stage-specific cocktail offers a promising approach towards the development of a malaria vaccine ideally capable of preventing initial infection, the clinical manifestation as well as the transmission of the disease. To investigate the potential of such an approach we combined proteins and domains (11 in total) from the pre-erythrocytic, blood and sexual stages of *P*. *falciparum* into a cocktail of four different components recombinantly produced in plants. After immunization of rabbits we determined the domain-specific antibody titers as well as component-specific antibody concentrations and correlated them with stage specific *in vitro* efficacy. Using purified rabbit immune IgG we observed strong inhibition in functional *in vitro* assays addressing the pre-erythrocytic (up to 80%), blood (up to 90%) and sexual parasite stages (100%). Based on the component-specific antibody concentrations we calculated the IC_50_ values for the pre-erythrocytic stage (17–25 μg/ml), the blood stage (40–60 μg/ml) and the sexual stage (1.75 μg/ml). While the results underline the feasibility of a multi-stage vaccine cocktail, the analysis of component-specific efficacy indicates significant differences in IC_50_ requirements for stage-specific antibody concentrations providing valuable insights into this complex scenario and will thereby improve future approaches towards malaria vaccine cocktail development regarding the selection of suitable antigens and the ratios of components, to fine tune overall and stage-specific efficacy.

## Introduction

Despite years of intensive research malaria still affects millions of people worldwide and claims more than 500,000 lives per year, predominantly in sub-Saharan Africa [1]. Many strategies have been pursued to develop efficient vaccine formulations that prevent infection or at least the clinical development of malaria, including peptides [2], non-replicating sporozoites [3], DNA vaccines [4], vectored and prime-boost vectored vaccines [5], various formulations of recombinant proteins and fusion proteins [6–9] and fusion peptides targeting different stages of the *Plasmodium falciparum* life cycle [10]. Sporozoite-based approaches have the potential to induce sterile immunity but manufacturing costs are high and distribution in developing countries is challenging [11].

Most protein-based vaccine candidates, like RTS,S, which have entered clinical trials, did not fulfill expectations [12]. Whereas RTS,S addresses the pre-erythrocytic stage of the parasite to prevent infection, other vaccine candidates like *Pf*AMA1 target the blood stage to reduce or prevent clinical manifestation. So-called transmission-blocking vaccines that target sexual-stage antigens and prevent transmission by the mosquito host are an essential add-on to protective or therapeutic vaccines in the battle to eradicate malaria [13]. As a potential fourth class of malaria vaccines anti-toxin vaccines have been proposed by Schofield et al. in 2002 [14]. A 1993 study with a multi-stage, multi-component malaria vaccine cocktail featuring pre-erythrocytic, blood, and sexual stage antigens yielded promising results [15] although further clinical development of this approach was not reported. Despite the underlying complexity associated with the development and regulatory approval of cocktails with multi-stage functionality, we believe that an optimal malaria vaccine must efficiently target at least these three stages of the parasite life cycle, and this should be the long-term goal in future malaria vaccine development as long as there is no effective way to induce sterile immunity using pre-erythrocytic vaccine candidates.

With the aim to include several promising antigens and domains (based on previously reported results of malaria vaccine development research) from the pre-erythrocytic, blood, and sexual stages of the parasite in a small number of recombinant proteins, we selected 10 antigens (*Pf*CSP_TSR [16], *Pf*CelTos [17–19], *Pf*TRAP_TSR [20], *Pf*MSP1_19_EGF1 [21], *Pf*AMA1 [22], *Pf*MSP4_EGF [23], *Pf*MSP8_EGF1 and EGF2 [24], *Pf*MSP3 [25], *Pfs*25 [26] and *Pfs*230 [27]) and combined them in three stage-specific fusion proteins (CCT: pre-erythrocytic stage: *Pf*CSP_TSR, *Pf*CelTos and *Pf*TRAP_TSR; E3: blood stage: *Pf*MSP1-19_EGF1, *Pf*MSP4_EGF, *Pf*MSP8_EGF1, *Pf*MSP8_EGF2 and *Pf*MSP3; F0: sexual stage: *Pfs*25 and *Pfs*230) together with gAMA1 (*Pf*AMA1, included separately to avoid an overly large fusion protein) as one of the leading blood stage antigens, to be used as a multi-component multi-stage vaccine cocktail called PlasmoMix. The final design of the three fusion proteins was a result of an iterative expression screening approach (using the transient plant expression system) aiming to combine previously described antigens or antigen domains in the context of a stable, well-expressing vaccine candidate antigen. All four proteins were produced by transient expression in *Nicotiana benthamiana* plants, a rapid and versatile production system that could be used for the cost-efficient manufacture of vaccines for developing countries [28, 29].

## Material and Methods

### Ethics statement

Rabbits were housed, immunized and sampled by Biogenes GmbH (Berlin, Germany), according to national animal welfare regulations. The animal facilities and protocols were reviewed and approved by: Landesamt fμr Landwirtschaft, Lebensmittelsicherheit und Fischerei MecklenburgVorpommern (LALLF M-V) (Approval No: 7221.3-2-030-13). To isolate the blood after immunization according to national regulations the animals were anesthetized using Ventranquil, stunned using a captive bolt device and exsanguinated by throat cut.

Primary human liver cells were freshly isolated from healthy remnant material following tumor removal surgery. The samples were received in an anonymized fashion and none of the authors or personnel on the study was involved in the anonymization. The material was destroyed after use in accordance with Dutch ethical legislation as described in the Medical Research (Human Subjects) Act. The guidelines issued by the ethics committee of the Radboud University Nijmegen Medical Center (full committee name: Commissie Mensgebonden Onderzoek) state that no informed consent is needed for studies that use anonymised materials and that do not reveal information that can be correlated to specific individuals or groups. The protocol used in the current study was reviewed by the ethical committee and the waiver for informed consent was confirmed by the committee.

### Construct design and cloning

**Fig 1:**
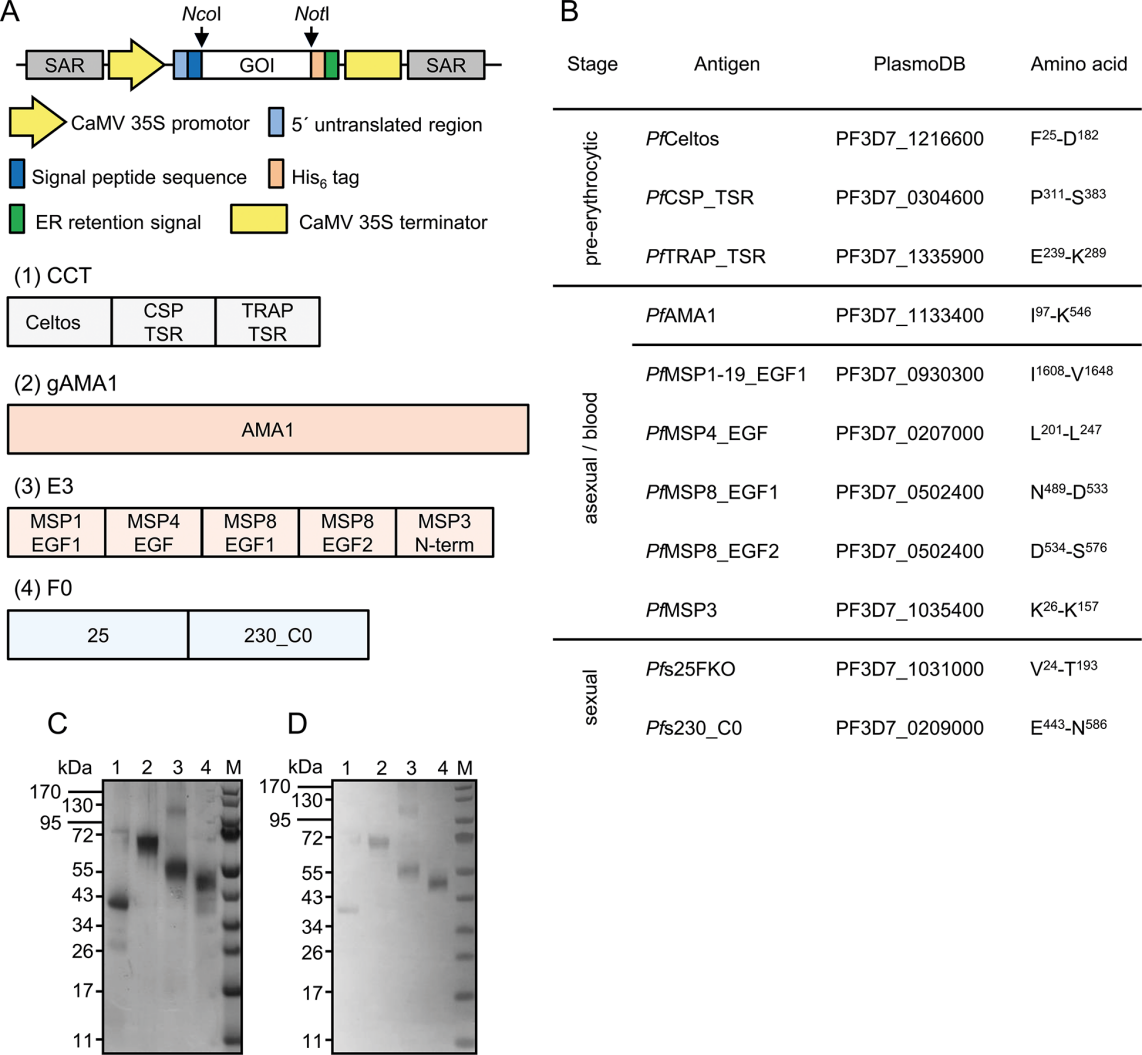
Plant expression cassette and construct design, amino acid sequences, SDS-PAGE and immunoblot blot analysis. (A) Schematic presentation of the expression cassettes of the plant binary expression vector pTRAk. SAR: scaffold attachment region; CaMV 35S promoter and terminator: promoter with duplicated enhancer and terminator of the *Cauliflower mosaic virus* (CaMV) 35S gene; 5' untranslated region: 5'-UTR of the chalcone synthase gene from *Petroselinum crispum*; signal peptide sequence: transit peptide sequence of murine antibody heavy chain; GOI: Gene of interest, CCT (1), gAMA1 (2), E3 (3) and F0 (4). The restriction sites used to insert the GOI into the plant expression vector are indicated; His_6_ tag: six histidine affinity purification tag; ER-retention signal: SEKDEL ER-retention signal. **(**B) Table containing all the information for the selected antigens. For each antigen the main stage of expression, the name, the plasmoDB number and the amino acid sequence are depicted. SDS-PAGE (C) and immunoblot analysis (D) under reducing conditions of the four recombinant and purified proteins. Proteins were detected using rabbit anti-His_6_ antiserum and alkaline phosphatase-labeled goat anti-rabbit antiserum. M: PageRuler pre-stained protein ladder (Fermentas), lane 1: CCT, lane 2: gAMA1, lane 3: E3 and lane 4: F0.

All cDNAs (Fig 1A and 1B) were obtained as *N*. *benthamiana* codon-optimized synthetic genes from Geneart (Invitrogen, Carlsbad, CA). The four constructs were cloned as described by Boes et al. [30] and Voepel et al. [31]. For antibody titer determination, the cDNAs coding for the single domains were fused to the C-terminus of the fluorescent reporter protein DsRed. The p19 silencing inhibitor gene (p19si) was modified as described [30]. All cloning steps were confirmed by DNA sequencing.

### Transient expression in *N*. *benthamiana*

The transformation and cultivation of *Agrobacterium tumefaciens* as well as the transient expression in *N*. *benthamiana* plants was performed as previously described by Boes et al. [30] and Voepel et al. [31].

### Purification of antigens

Infiltrated *N*. *benthamiana* leaves were harvested 5 days post infiltration and ground in liquid nitrogen. Purification of gAMA, F0 and CCT was performed as described by Boes et al. [30], Beiss et al. [32] and Voepel at al. [31] respectively. E3, and all DsRed fusion proteins were purified according to the procedure described by Voepel et al. [31] with modified extraction buffers. For the DsRed fusion proteins, we used PBS plus 500 mM NaCl, pH 7.4. For E3, we used 50 mM Tris-HCl pH 8 plus 500 mM NaCl.

### SDS-PAGE and immunoblot analysis

Proteins were separated on 4–12% (w/v) polyacrylamide gradient gels (NuPage, Life Technologies) and either stained with Coomassie Brilliant Blue or transferred onto a nitrocellulose membrane (Whatmann, Dassel, Germany) for immunoblot analysis as previously described by Boes et al. [30].

### Rabbit immunization, antibody titer determination and IgG purification

Three rabbits were immunized with the vaccine cocktail comprising equal amounts of the four components formulated with a proprietary Biogenes adjuvant (an oil in water emulsion containing lipopolysaccharides) on days 0, 7, 14, 28, 49 and 77, using 200 μg of antigen mix for the prime immunization and 100 μg of antigen mix for the consecutive boosts. Serum samples were taken on days 35 (bleed 1), 63 (bleed 2) and 91 (bleed 3). Antibody titer determination and IgG purification was performed as previously described by Boes et al. [30].

### Calibration free concentration analysis (CFCA)

Antigen-specific antibody concentrations were measured in the purified antibody preparations by CFCA [33] using a Biacore T200 instrument. The four purified components (CCT, gAMA1, E3 and F0) were individually covalently coupled to CM5-S-Series sensor chips by standard EDC-NHS chemistry. The method is described in detail by Boes et al. [30].

### Surface plasmon resonance (SPR)-based competition assay

For the reversal of growth inhibition experiments, we determined the concentrations of E3 and gAMA1 that completely blocked the E3-specifc and gAMA1-specifc antibodies. The antigens were buffer exchanged against RPMI 1640 (PAA, E15-041) with HEPES without L-glutamine by repeated rounds of dilution and concentration using a VivaSpin 15R column with a MWCO of 10 kDa. Total IgG preparations from the third bleed (day 91) were diluted to a final concentration in the competition mix resulting in 40–70% inhibition in a standard growth inhibition assay, and incubated overnight at 4°C with either gAMA1 (final concentrations 120, 12 and 1.2 μg/ml) or E3 (final concentrations 1230, 123 and 12.3 μg/ml). The competition mix was analyzed against the corresponding antigen (E3 or gAMA1) immobilized on the CM5-S-Series sensor chips using a Biacore T200 instrument. Full competition was defined as a RU signal less than 5% of the sample containing no competitor.

### Immunofluorescence assay (IFA)

Indirect IFAs with IgG (final concentration 1.5 μg/ml) purified from PlasmoMix-specific rabbit immune sera were carried out using sporozoites, schizonts, macrogametes and retorts. of *P*. *falciparum* strain NF54 as previously described by Boes et al. [30].

### Inhibition of sporozoite gliding motility (SGM), hepatocyte cell traversal (HCT) and sporozoite invasion and liver stage development (SILSD) assays

The isolation of NF54 sporozoites and their use in the gliding motility and cell traversal assays were carried out as described by Behet et al. [34], with adaptation that for scoring the gliding motility the number of gliding circles of 40 trails in 2 distinct wells was counted. The SILSD assay was performed as described [35] with the following adaptations. For each well, sporozoites were pre-incubated with rabbit IgG for 30 min on ice, after which the sporozoite-antibody mixture was transferred onto the hepatocytes. On day 4 after sporozoite invasion, cells were washed three times with PBS, fixed with 4% paraformaldehyde for 15 min at room temperature and stained extracellular with anti-*Pf*CSP-FITC or for the dose response experiment with anti-*Pf*MSP1 followed by goat anti-mouse A488 as well as intracellular with anti-*Pf*HSP70-biotin (StressMarq) followed by streptavidin-AF647 (Invitrogen) and 4',6-diamidino-2‐fenylindool (DAPI). The numbers of intracellular mature parasites were determined in 20 fields by microscopy at 200x magnification. In the dose response experiments 16 fields at 100x magnification (*Pf*HSP70) or 25 fields at 200x magnification (*Pf*MSP1) were analyzed.

### *In vitro* growth inhibition assay (GIA)

The ability of polyclonal rabbit IgGs to inhibit the growth of *P*. *falciparum* 3D7A was determined using growth inhibition assays (GIAs) as previously described by Boes et al. [30].

### Reversal of growth inhibition assay

A reversal of growth inhibition assay was used to determine the ability of antibodies directed against either gAMA1 or E3 to inhibit parasite growth and to assess their contribution to the overall inhibitory capacity of the immune serum. Antibodies from rabbits R1, R2 and R3 purified from immune sera collected on day 91 were mixed with component E3 (final concentration 24 μg/ml) to completely block E3-specific antibodies. The mixture was incubated overnight at 4°C and subsequently used for a GIA as described above. The contribution of gAMA1-specific antibodies was assessed in the same way by adding gAMA1 (final concentration 120 μg/ml) to the purified antibodies. The amount needed to completely block the antibodies was determined using the SPR-based competition experiment described above. In the absence of antigen, the respective antibody concentration resulted in 40–70% inhibition in a standard GIA.

### Antibody-dependent respiratory burst (ADRB) assay

The stimulation of human polymorphonuclear neutrophil granulocytes (PMNs) was analyzed in ADRB assays in a total volume of 75 μl as described [36], using samples from rabbit R3_91 as example. gAMA1 or E3 (750 ng) were immobilized for the solid-phase ADRB (sADRB) assay. Purified antibodies were adjusted to 2.5 mg/ml total IgG (comparable to the amounts used in the reversal of growth inhibition assay). Purified IgG from normal rabbit serum (NRS) was used at same concentration. The stimulation of 7.5 x 10^4^ PMNs was determined by luminol-enhanced chemiluminescence.

### Standard membrane feeding assay (SMFA)

Standard membrane feeding assays [37] were used to determine the ability of purified rabbit anti-PlasmoMix antibodies to block the transmission of *P*. *falciparum* from human to mosquito as previously described by Feller et al. [38]. Antibodies purified from rabbit immune sera were used at 1 mg/ml total IgG. The IC_50_ value was determined by analyzing purified antibodies from day 91 at concentrations of 1, 0.1 and 0.01 mg/ml total IgG. Based on the CFCA results, the total IgG concentration was converted to the F0-specific antibody concentration and plotted against the inhibition of transmission. Statistical analysis (Mann-Whitney U) was carried out for median numbers of oocysts between groups of mosquitoes receiving either antibodies purified from normal rabbit serum (NRS) or rabbit immune sera. The oocyst prevalence between groups of mosquitoes either receiving antibodies purified from NRS or rabbit immune sera was statistically analyzed using Fisher’s exact test.

## Results

### Production of recombinant antigens

*A*. *tumefaciens* strains carrying the component expression constructs in the context of the binary expression vector pTRA (Fig 1A and 1B) were vacuum infiltrated into *N*. *benthamiana* plants, and subsequent purification of the recombinant antigens CCT (*Pf*CSP_TSR, *Pf*CelTos and *Pf*TRAP_TSR), gAMA1(*Pf*AMA1), E3 (*Pf*MSP1-19_EGF1, *Pf*MSP4_EGF, *Pf*MSP8_EGF1, *Pf*MSP8_EGF2, *Pf*MSP3) and F0 (*Pfs*25, *Pfs*230) from crude tobacco extracts yielded highly pure and intact proteins of the expected sizes, as shown by reducing SDS-PAGE and immunoblot (Fig 1C and 1D). Yields were > 1 mg/g leaf tissue for CCT and gAMA1 and > 250 μg/g leaf tissue for E3 and F0.

### Component and domain-specific antibody titers

**Fig 2:**
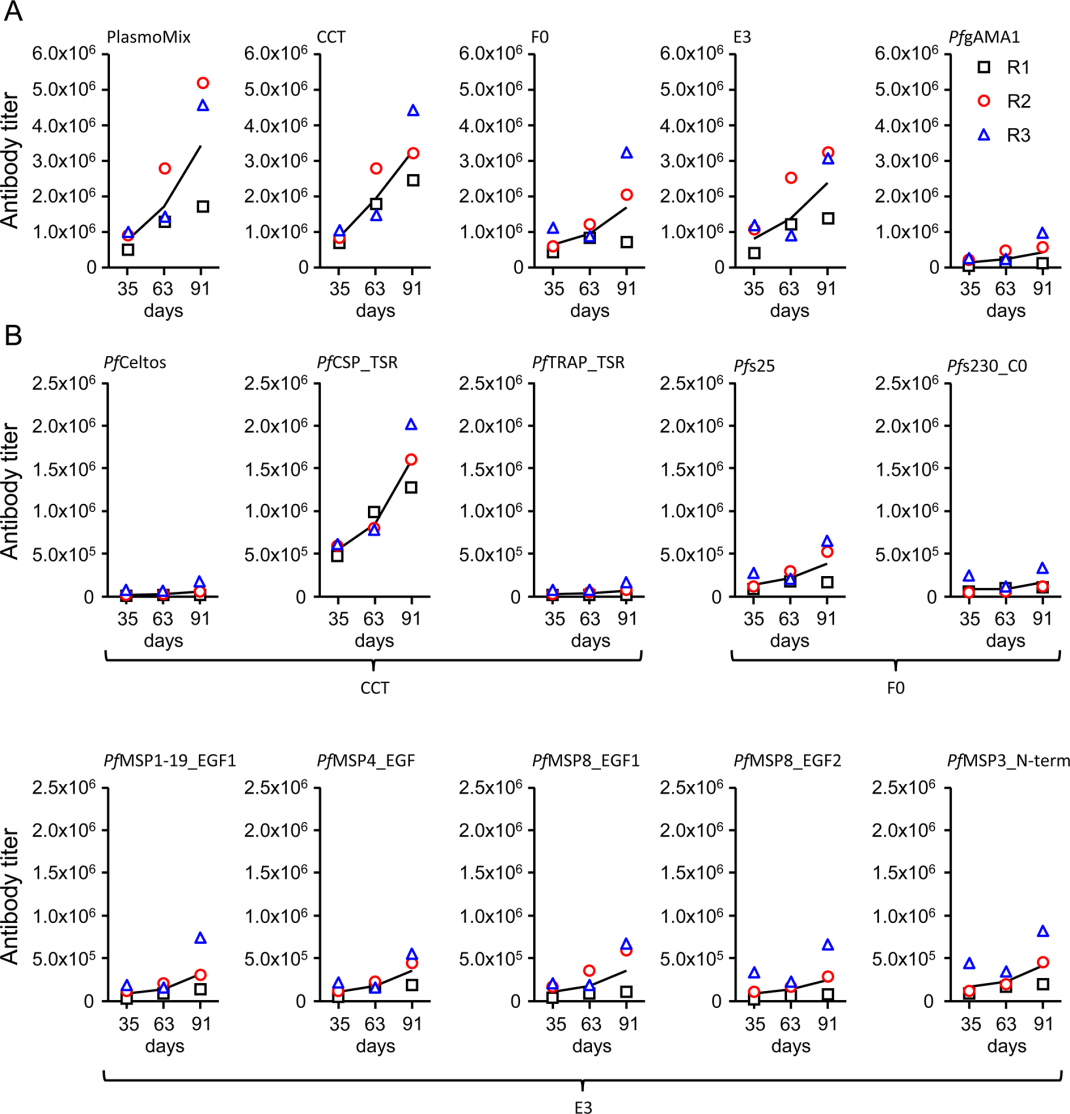
Antigen-specific antibody titers of rabbit immune sera determined by ELISA. Three rabbits (R1, R2 and R3) were immunized six times with PlasmoMix and serum samples were collected on days 0 (pre-immune), 35, 63 and 91. (A) The antibody titer against the immunization mixture (PlasmoMix) as well as against the four protein-based components (CCT, E3, gAMA1 and F0) were determined. (B) To further dissect the immune response, the specific antibody response against each individual domain was analyzed. Therefore, the domains were C-terminaly fused to DsRed (fluorescent reporter protein), and expressed and purified as described in the methods section. Antigen domains comprising a fusion protein are connected by a bracket. Antibody titers are shown for each rabbit (R1: open black square, R2: open red circle, R3: open blue triangle) as well as the geometric mean (black line) of three rabbits.

Antibody titer analysis was performed for the cocktail (PlasmoMix), the components (CCT, F0, E3 and gAMA1) as well as the individual recombinant domains. Antibody antibody titers were monitored at day 0, 35, 63, and 91, and an increase of antibody titers over the sampling period was observed for all tested antigens and domains. We recorded a antibody titer of 3.8 x 10^6^ against the cocktail, antibody titers of 1.9–3.3 x 10^6^ against CCT, F0 and E3, and a antibody titer of just below 5.5 x 10^5^ against gAMA1 (Fig 2A). For CCT, we observed a strong bias in the antibody titers against the three pre-erythrocytic stage antigens *Pf*Celtos, *Pf*CSP_TSR and *Pf*TRAP_TSR, a antibody titer of 1.6 x 10^6^ against the TSR-domain of *Pf*CSP and 20-fold lower values for the other two domains (Fig 2B). In contrast, all five blood-stage domains within E3 induced comparable antibody titers of approximately 4.0 x 10^5^. A two-fold difference in antibody titers was observed when we compared the transmission blocking-component F0 (2 x 10^6^) with the individual antibody titers of the two sexual-stage antigens at 4.4 x 10^5^ for *Pf*s25 and 1.8 x 10^5^ for *Pf*s230_C0 (Fig 2B).

### Quantification of antigen-specific IgG responses

We performed SPR-based CFCA for the precise quantitation of the component-specific antibodies in the IgG preparations purified from rabbit immune sera that were subsequently used in *in vitro* inhibition assays to determine IC50 requirements for stage-specific antibody concentrations. Table 1 shows component-specific antibody concentration given as mg/ml as well as % total IgG. Addintionally Plasmomix-specific IgG concentrations were calculated by adding up the values obtained for the 4 components. Up to 12% (of total IgG) of Plasmomix-specific antibodies was observed for rabbit R2 in the 63 day sample.

**Table 1:** The purified antibody preparations used for all *in vitro* assays. The total IgG concentrations and CFCA results are listed for each component. Nomenclature is based on the number of the rabbit (R1, R2 or R3) followed by the sampling day (35, 63 or 91). The PlasmoMix-specific antibody concentration is the sum of the specific antibody response against the four components. The antigen-specific antibody concentration is given in mg/ml and % of total IgG.

Sample	total IgG	PlasmoMix-specific-IgG		CCT-specific-IgG		gAMA1-specific-IgG		E3-specific-IgG		F0-specific-IgG	
	mg/ml	mg/ml	% total IgG	mg/ml	% total IgG	mg/ml	% total IgG	mg/ml	% total IgG	mg/ml	%total IgG
R1_35	21.70	1.23	5.65	0.40	1.85	0.41	1.87	0.16	0.76	0.26	1.18
R1_63	21.18	1.88	8.85	0.68	3.19	0.51	2.41	0.36	1.70	0.33	1.56
R1_91	19.09	1.19	6.25	0.50	2.59	0.26	1.34	0.25	1.30	0.20	1.02
R2_35	22.60	1.72	7.63	0.31	1.35	0.75	3.32	0.38	1.69	0.29	1.26
R2_63	17.08	2.17	12.69	0.50	2.90	0.84	4.92	0.47	2.77	0.36	2.11
R2_91	22.25	2.34	10.52	0.63	2.83	0.78	3.51	0.54	2.43	0.39	1.75
R3_35	27.40	2.24	8.17	0.31	1.13	0.80	2.90	0.52	1.89	0.62	2.25
R3_63	23.13	1.46	6.30	0.28	1.21	0.51	2.20	0.29	1.26	0.38	1.62
R3_91	25.03	1.95	7.79	0.54	2.16	0.62	2.46	0.41	1.62	0.39	1.56

### IgG reactivity with parasite surface antigens in immunofluorescence analysis and schizont lysate immunoblot analysis

The ability of the antigen cocktail to induce antibodies recognizing *P*. *falciparum* surface proteins in their native context was confirmed by testing the purified IgG fractions against parasites from different life cycle stages (sporozoites, schizonts, macrogametes and retorts) in immunofluorescence assays. We observed strong staining localized at the surface of all *P*. *falciparum* preparations (S1 Fig). No staining was observed for the NRS negative control. Additionally the reactivity against the native blood-stage antigens included in the context of PlasmoMix was confirmed by immuno blot analysis using schizont lysate and purified immune IgG from R1, R2 and R3 (Day 91). As shown in S2 Fig all *Plasmodium falciparum* blood-stage proteins included within PlasmoMix could be identified based on both, molecular weight and data reported by others: putative high molecular weight *Pf*MSP-complex, unprocessed *Pf*AMA1 or *Pf*MSP8, processed *Pf*AMA1, *Pf*MSP3, processed *Pf*MSP1_42, *Pf*MSP4 and processed *Pf*MSP1_19 or processed *Pf*MSP8.

### Analysis of *in vitro* pre-erythrocytic stage efficacy by inhibition of sporozoite gliding motility (SGM), hepatocyte cell traversal (HCT) and sporozoite invasion and liver stage development (SILSD)

**Fig 3:**
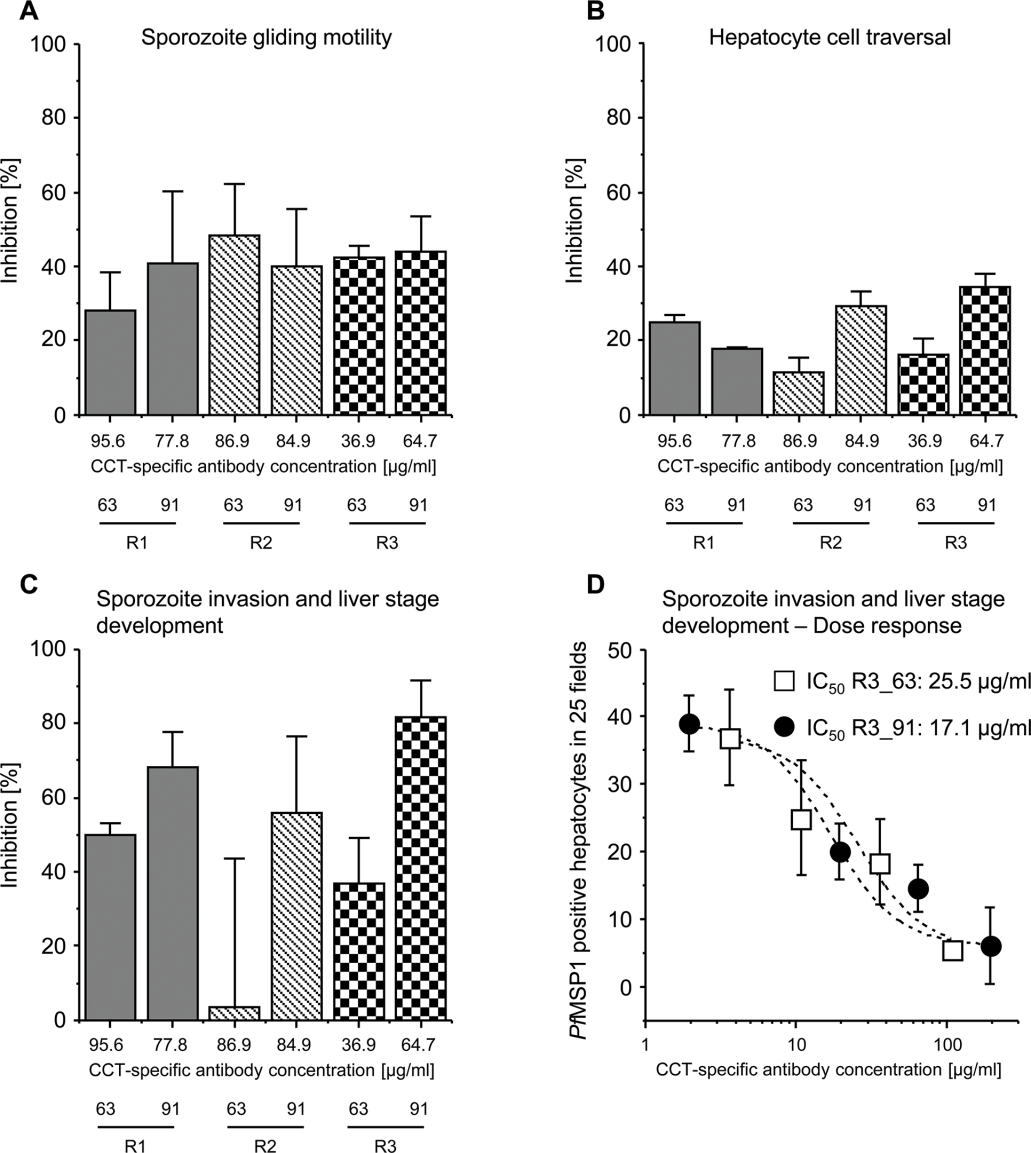
*In vitro* inhibition of (A) sporozoite gliding motility, (B) hepatocyte cell traversal, (C) sporozoite invasion and liver stage development with PlasmoMix-specific rabbit IgG and (D) Dose response of sporozoite invasion and liver stage development. All three assays were performed with *P*. *falciparum* NF54 parasites and purified rabbit IgGs (R1, R2 and R3) at a total IgG concentration of 3 mg/ml from serum samples collected on days 63 and 91. For the dose response, purified rabbit IgGs from rabbit R3 (day 63 and day 91) was used at the following total IgG concentrations: 9, 3, 0.9 and 0.09 mg/ml. The CCT-specific antibody concentration was calculated based on CFCA from total IgG. Inhibitions are expressed as the mean of duplicate measurements with standard deviations.

The inhibitory efficacy of the pre-erythrocytic stage components in the context of the fusion protein CCT was investigated using PlasmoMix-specific rabbit immune IgG in SGM, HCT and SILSD assays. IgG samples from all three rabbits were tested in duplicate at a final concentration of 3 mg/ml and we observed up to 48% inhibition of SGM, 34% inhibition of HCT and 81% inhibition of SILSD (Fig 3). Further, IC_50_ values for the inhibition of SILSD were determined in a dose response experiment using purified immune IgG from rabbit R3 at day 63 and 91 (R3_63: 25.5 μg/ml and R3_91: 17.1 μg/ml).

### Analysis of *in vitro* blood stage efficacy by growth inhibition assay (GIA)

**Fig 4:**
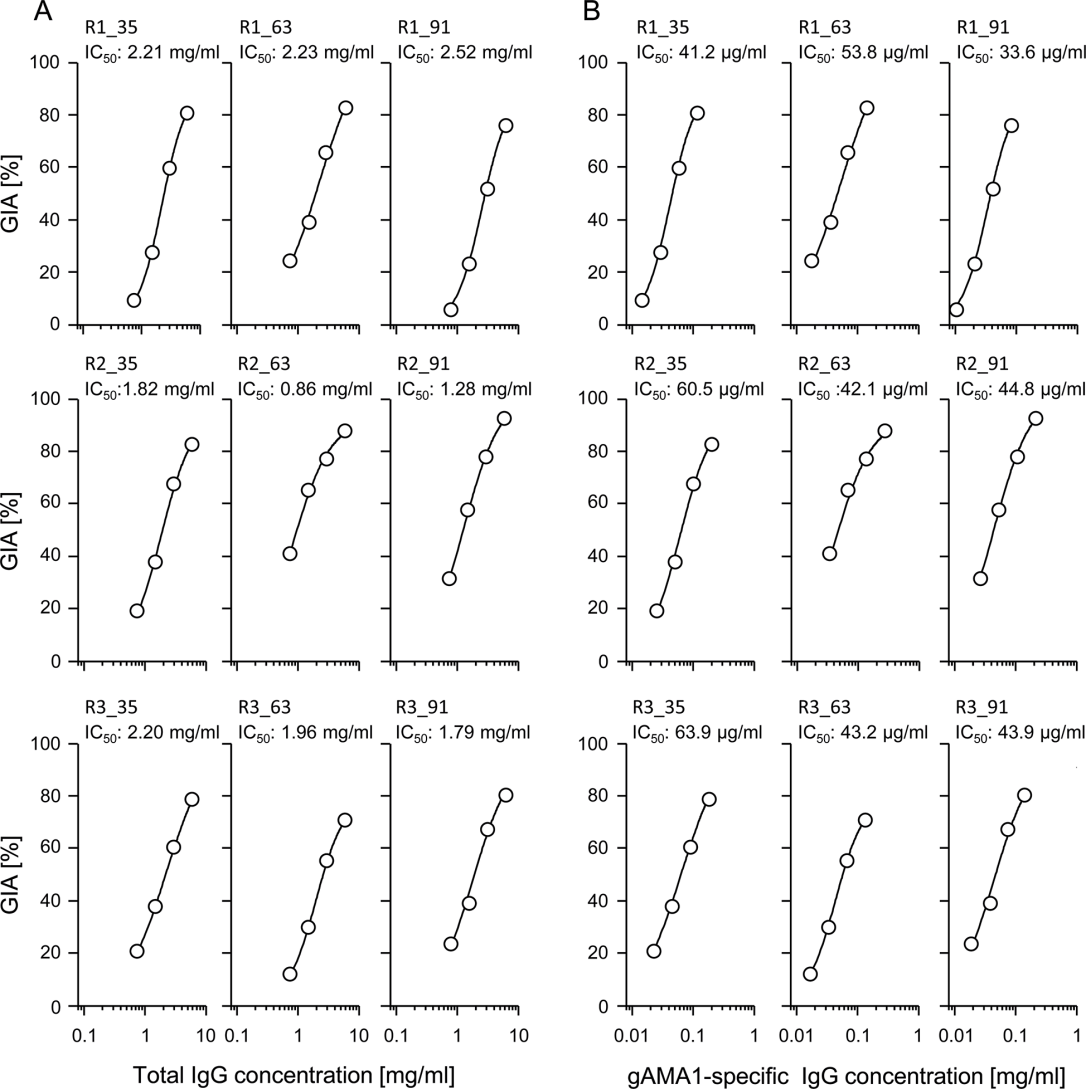
*In vitro* growth inhibition assay (GIA) of asexual *P*. *falciparum* 3D7 parasites with purified PlasmoMix-specific rabbit IgG. The rabbit IgGs were purified from the serum of three rabbits (R1, R2 and R3) collected on days 35, 63 and 91 post-immunization with PlasmoMix. Nomenclature of the sample first features the number of the rabbit (R1, R2 or R3) followed by the sampling day (35, 63 or 91). (A) Four serial 1/1 dilutions from 6–0.75 mg/ml of total IgGs were used to demonstrate the concentration dependency of the assay and to calculate the IC_**50**_ values (the total IgG concentrations needed for 50% inhibition). (B) The same GIA but instead of total IgG, the gAMA1-specifc antibody concentration (based on CFCA and calculated from total IgG, see methods section) was used and the IC_**50**_ values for gAMA1-specific antibodies were calculated. Each data point represents the mean of technical triplicates.

**Fig 5:**
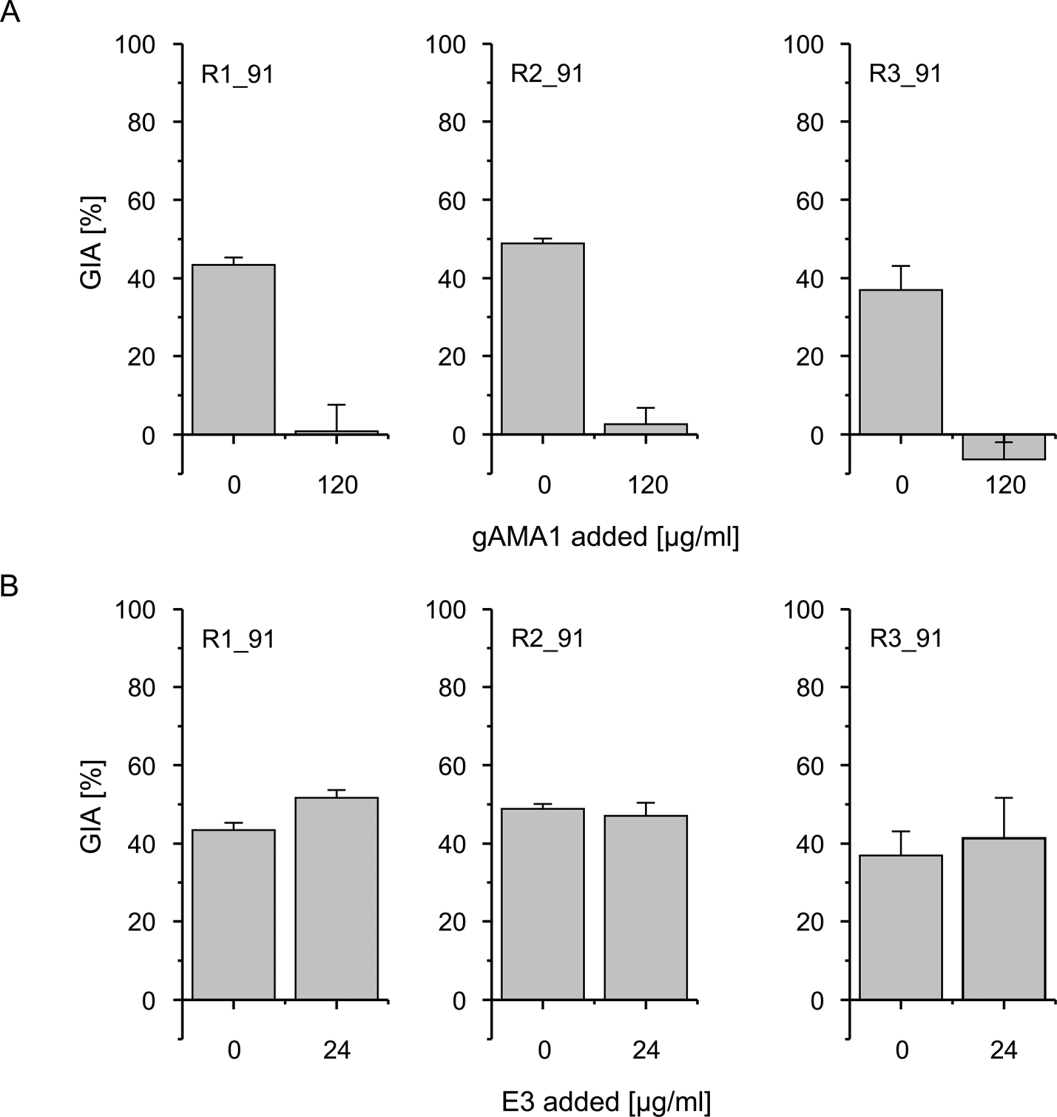
Reversal of growth inhibition assay. The assays were performed with *P*. *falciparum* 3D7 parasites and with the intention to assess the inhibitory potential of antibodies directed against either gAMA1 or E3. The GIA was run with purified antibodies from serum samples taken on day 91 post-immunization with PlasmoMix. The assay was carried out with a total IgG concentration resulting in 40–70% inhibition in a standard GIA in the presence of either gAMA1 (A) or E3 (B) to reverse the inhibition. Each data point represents the mean with standard deviation of technical triplicates.

The efficacy of the blood-stage component of the vaccine cocktail (antigens gAMA1 and E3) was assessed using a classical GIA. Up to 90% inhibition was observed at a concentration of 6 mg/ml total IgG purified from rabbit immune sera (Fig 4A). Determination and comparison of IC_50_ values for antigen-specific IgGs indicated that the IC_50_ values for gAMA1-specific antibodies (Fig 4B) were in the same range (30–60 μg/ml) as reported in a previous study [30] after the immunization of rabbits with recombinant plant-derived gAMA1, indicating that the growth inhibition induced by the cocktail is mainly mediated by gAMA1-specific antibodies. To precisely quantify this contribution, a reversal of growth inhibition assay was performed by competitive neutralization of these antibodies with recombinant gAMA1 and E3 (Fig 5). Although growth inhibition was completely abolished by 120 μg/ml gAMA1, the neutralization of E3-specific IgGs using 24 μg/ml E3 did not affect growth inhibition.

### Analysis of *in vitro* sexual stage efficacy by standard membrane feeding assay (SMFA)

**Fig 6:**
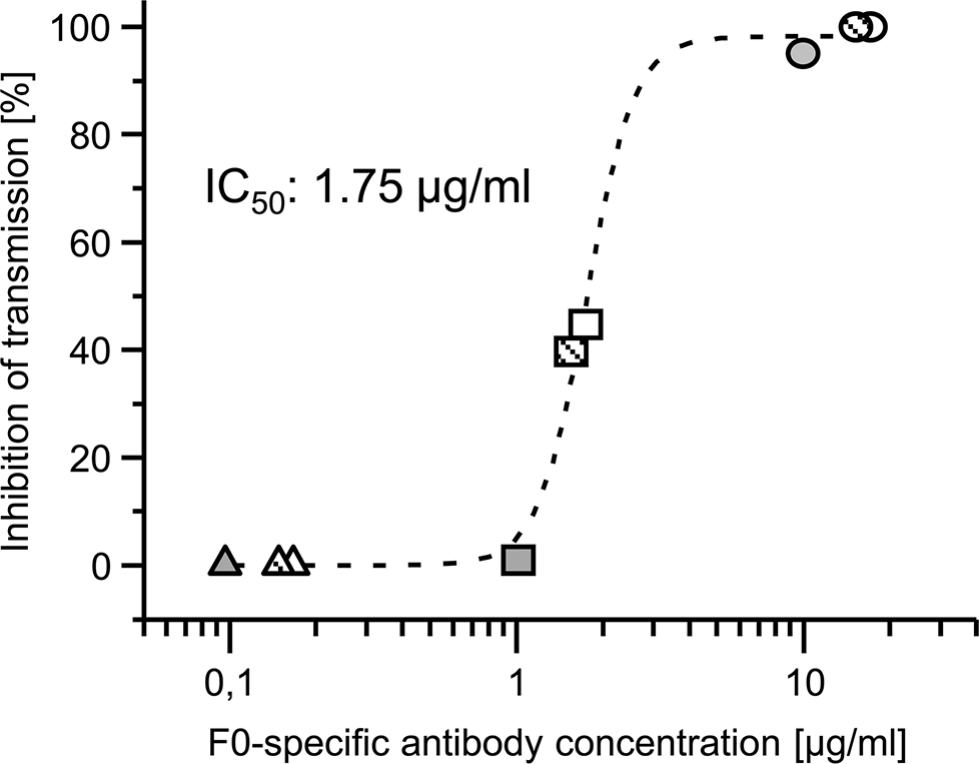
Correlation of transmission blocking activity and F0-specific antibody concentration. The rabbit IgGs were purified from serum samples collected on day 91 post-immunization with PlasmoMix. To demonstrate the concentration dependency of the transmission blocking activity, the TBA was performed with purified antibodies from each rabbit (R1: black, R2: open and R3: hatched) at total IgG concentrations of 1 mg/ml (circles), 0.1 mg/ml (squares) and 0.01 mg/ml (triangles). Based on the CFCA results, the F0-specific antibody concentration was calculated, plotted and used to determine the IC_50_ value (the antibody concentration needed to obtain 50% inhibition of transmission). For detailed results of the transmission blocking assay, refer to S2 Table.

The SMFA was used to investigate the transmission-blocking efficacy of the sexual stage-specific component F0 (*Pf*s25 and *Pf*s230_CO). The observed transmission-blocking activity (reduction in oocyst formation) of 95–100% for the samples from the three animals for all three bleeds (S1 Table) demonstrated the potency of the F0 fusion antigen comprising *Pf*s25 and *Pf*s230_CO, agreeing with previous reports based on these antigens [15, 27, 39]. The amounts of F0-specific antibodies measured in the samples taken at day 91 by SPR-based CFCA analysis were correlated with a corresponding dose response TBA, revealing an IC_50_ value of < 2 μg/ml (Fig 6).

## Discussion

A cocktail featuring key antigens from different stages of the *P*. *falciparum* life cycle is a promising approach for malaria vaccine development. We therefore investigated the antibody responses generated by immunizing rabbits with an equal mixture of four different recombinant proteins featuring suitable antigens from the pre-erythrocytic (*Pf*CSP_TSR, *Pf*CelTos and *Pf*TRAP_TSR), blood (*Pf*AMA1, *Pf*MSP1-19_EGF1, *Pf*MSP4_EGF, *Pf*MSP8_EGF1, *Pf*MSP8_EGF2 and *Pf*MSP3), and sexual stages (*Pf*s25 and *Pf*s230) of *P*. *falciparum*. The four recombinant proteins were successfully produced by transient expression in *N*. *benthamiana* plants. Yields of 0.25–1.0 mg per g fresh leaf weight were comparable to typical expression levels achieved using this system [40, 41] and the fact that intact and highly pure proteins could be isolated indicated that all four vaccine antigens are suitable for production in the plant transient expression system.

The analysis of PlasmoMix-specific rabbit immune sera obtained using a hyperimmunization protocol that aims for maximum antibody titers revealed differences in reactivities against the different antigen domains with antibody titers ranging from 7.7 x 10^4^ (*Pf*Celtos and *Pf*TRAP) to 1.6 x 10^6^ (*Pf*CSP-TSR). The observed antibody titer bias between fused domains as observed for CCT and F0 agrees with our previously observed data based on corresponding mouse immune sera [31, 32]. The gAMA1-specific antibody titers were 2–3 times lower than we previously observed for gAMA1-specific rabbit immune sera, where 3.5 times more antigen was used [30]. The antibody titers of the domains within the E3 component were balanced at 3.5 x 10^5^. Immunofluorescence analysis confirmed the reactivity of the PlasmoMix-specific immune sera against all the tested *P*. *falciparum* stages, and the staining patterns were in accordance with the results of previous studies by our group and others [30, 31, 39, 41–43] after immunization with the same or comparable antigens. Typical *Pf*AMA1-specific apical staining of merozoites could not be observed, since many other non-localized merozoitze surface proteins were stained in parallel. To further confirm the reactivity against all individual blood-stage antigen included in PlasmoMix a schizont lysate immunoblot was performed with purified PlasmoMix-specific rabbit immune IgG. The detected bands were identified by apparent molecular weight in comparison with the following studies performed on the respective *Plasmodium falciparum* proteins [44–48].

For ethical reasons and because of these preexisting results for single antigen vaccination experiments with three of the PlasmoMix components we did not perform single antigen vaccinations for all the four components along with the PlasmoMix experiments. In this context it should be noted that this initial study to investigate a multistage vaccine cocktail was not performed with a human compatible adjuvant or vaccination regime and that conclusions regarding the effect of such a cocktail under different conditions have to be further investigated.

Stage-specific inhibition assays were used to address the *in vitro* efficacy of PlasmoMix. The three pre-erythrocytic stage assays showed promising inhibition values between 34% for hepatocyte cell traversal and 81% for hepatocyte invasion and maturation. These values are high compared to our previous results using CCT in a mouse immunization study, which achieved a reduction of only 35% in a hepatocyte invasion assay [31]. However these results were obtained using the immortalized liver cell line HepG2 rather than the human primary hepatocytes used in this study. R3 is the only rabbit that showed a correlation between the concentration of CCT-specific IgG and the percentage effect in both the HCT and SILSD assays, but not the SGM assay, over time. There was no link between overall CCT IgG-specific antibodies and specificity, which might indicate that each rabbit produces different amounts of functional IgG. Clear inhibition in pre-erythrocytic *in vitro* assays has been reported for the single antigens *Pf*CSP_TSR and *Pf*Celtos in previous studies [19, 49], whereas the results for *Pf*TRAP are contradictory [20, 50]. Antibodies against *Pf*CSP play a role in gliding motility [51], whereas *Pf*Celtos-specific antibodies interfere with cell traversal and have another mode of action affecting not only sporozoites at the site of infection but also intra-hepatocyte parasites [52]. We believe that the inhibition observed in the three different assays could be a cumulative effect of all three antigens and further studies are needed to dissect the contribution of each antigen. The IC_50_ values determined for the rabbit R3 (R3_63 & R3_91) were in the lower μg/ml range (17.1–25.5 μg/ml) and therefore in between the IC_50_ value observed for the sexual-stage and blood-stage specific inhibition assays (see below). It is difficult to compare the IC_50_ value with data from other studies, because most of them use ELISA units (EU) or arbitrary units (AU) instead of absolute concentrations of antigen-specific IgG as we have determined by CFCA in this study. Estimations on protective levels of *Pf*CSP-specific antibodies derived from immunization studies with RTS,S suggest concentrations of > 20μg/ml [53].

Up to 90% inhibition was observed in the GIA with 6 mg/ml total IgG. Surprisingly, as shown by the reversal of growth inhibition, the activity in this experimental context was exclusively mediated by anti-*Pf*AMA1 antibodies. This conclusion is also supported by the IC_50_ values for gAMA1-specific antibodies, which provide no evidence for a synergistic or additive effect of E3-specific antibodies in this GIA when compared to similar values induced by gAMA1 alone [30]. At least *Pf*MSP1-19-specific antibodies play a role in naturally acquired immunity [54] and immunization with recombinant *Pf*MSP1-19 can induce growth inhibitory antibodies [55] even though it is known that *Pf*AMA1 is more immunogenic and causes stronger growth inhibition than *Pf*MSP1-19 [56]. Faber et al. [8] demonstrated a small but significant contribution of *Pf*Msp1-19-specific antibodies induced by immunization with *Pf*AMA1-DICO_*Pf*Msp1-19 fusion proteins. In this study, an IC_50_ of >200 μg/ml *Pf*Msp1-19 specific antibodies (affinity purified from rabbit immune IgG) was determined, leading us to the conclusion that the lacking effect of E3-specific and/or *Pf*Msp1-19-specific IgGs might be explained by the much lower concentrations of these antibodies (<50 μg/ml for E3 and, looking at the antibody titer ratios, most probably less than 10 μg/ml for *Pf*MSP1-19) induced by the immunization with PlasmoMix. Our GIA data for blood-stage malaria vaccine candidate efficacy highlight the role of *Pf*AMA1 as a leading blood-stage antigen. Nevertheless, we included the fusion protein E3 to provide additional promising blood-stage antigens such as *Pf*MSP3 and *Pf*MSP1-19, which have been associated with antibody-dependent cell-mediated immunity (ADCI) [24,48], to diversify the blood-stage efficacy of the vaccine cocktail, even though we did not perform ADCI assays in the context of this work. Antibody-dependent respiratory burst assay (ADRB) is another method to address parasite-neutralizing activity of antibody preparations using human polymorphonuclear neutrophil granulocytes (PMN) as effector cells [36]. In the context of this work we conducted an initial experiment that demonstrates for the first time that rabbit antibodies can stimulate human PMNs (S3 Fig). We confirmed that gAMA1 as well E3-specific rabbit immune IgG are capable of inducing respiratory burst in a direct coating assay. In future studies we will develop and implement these assays to analyze ADRB activity of rabbit immune IgG on parasites.

Besides the theoretical ability to generate broadly-neutralizing immune responses, the use of cocktails containing several recombinant antigens bears the risk of diluting the response and losing efficacy by antigenic competition. In the context of PlasmoMix, we can address this hypothesis by combining the data reported herein with our previous results obtained after the immunization of rabbits with *Pf*gAMA1 [30]. While overall antibody titers directly correlated with the amounts of gAMA1 used in the PlasmoMix or gAMA1 immunizations, the inhibitory quality of the induced antibodies was not affected (similar IC_50_ values for the gAMA1-specific IgG). Anyhow in the cocktail a significant increase of the IC_50_ value for total IgG and thus a lower GIA efficacy was observed. Even though we did not generate comparable data for the remaining three PlasmoMix components we are convinced that antigenic competition plays a significant role in responses to multi-antigen cocktails. Although based on an observation with just one cocktail, this finding should be taken as a serious message that antigens within vaccine cocktails must be chosen carefully, and their number should be kept as low as possible to avoid the observed dilution of essential inhibitory antibodies.

The 100% reduction of oocyst formation observed in the sexual stage transmission blocking assay is in agreement with our own data from mouse immunizations with F0 [32] and results from previous studies with different variants of *Pf*s25 [26, 39, 40, 57] or *Pf*s230 [27, 41, 42]. The determined IC_50_ value (<2 μg/ml) is below, but in the same range as the one reported by ([58], 4.2μg/,ml) using *Pfs*25-specific rabbit immune IgG. The observed IC_50_ value may derive from both, *Pfs*25 and *Pfs*230_C0-specific IgG, anyhow the lack of replicates does not allow the conclusion that an additional contribution of *Pfs*230_C0-specific antibodies is the reason for the two fold lower value. Therefore it will be necessary to dissect and quantify the contribution of each of the sexual-stage antigens (Pfs25 and Pfs230_C0) in additional experiments to optimize the next generation of the PlasmoMix vaccine cocktail. The results also indicate that, at least in rabbits, it should be possible to reduce the amount of this component within the vaccine cocktail without compromising transmission-blocking efficacy, thus allowing other components to be over-represented and to induce higher antibody antibody titers for optimal protection against other (pre-erythrocytic and blood) stages.

## Conclusions

Our data suggest that cocktails comprising different stage-specific components remain a promising approach for the development of a malaria vaccine. However, our results also indicate the different components should not be mixed in equal amounts but in optimal ratios to generate what may be called “balanced efficacy”. The fine tuning of a new vaccine cocktail containing the PlasmoMix antigens may require: (1) the removal of some antigens; and (2) the adjustment of the relative concentrations of the remaining to compensate for the observed different IC_50_ requirements and at the same time taken into account the role of the respective stage in the context of the establishment and the clinical manifestation of the disease.

## Supporting Information

S1 Fig Immunofluorescence assay of NF54 parasites in different stages. For IFAs, P. falciparum NF54 parasites in the sporozoite, schizont, macrogamete and retort stages were fixed with methanol on the surface of a slide. Exemplary shown is the detection with purified IgGs from rabbit R2 from the serum sample collected on day 91 after immunization with PlasmoMix. As positive controls, murine antisera against *Pf*CSP (sporozoite), *Pf*MSP1-19 (schizonts), *Pfs*25 (magrogametes) and *Pfs*28 (retorts) were used, respectively. Rabbit antibodies were visualized with anti-rabbit secondary antibody labeled with Alexa Fluor 594 (red) while visualization of murine antibodies was performed with secondary Alexa Fluor 488 labeled anti-murine antibody (green). Parasite nulcei were highlighted with Hoechst 33342 (blue). Transm: Transmission light. Bar: 5 μm.
(TIF)

S2 Fig Schizont lysate immunoblot analysis. Purified rabbit immune IgG (R1, R2 and R3 from day 91) were used to detect native Plasmodium falciparum blood-stage antigens in immunoblot analysis with schizont lysate. 12 μl lysate (corresponding to 1.5x10^7^ schizonts) were separated under non-reducing conditions by SDS-PAGE. After immunoblot the membrane was blocked with skimmed milk and probed with rabbit immune IgG adjusted to 5 μg/ml *Pf*AMA1-spacific IgG concentration. Bound rabbit antibodies were detected with an alkaline phosphatase goat anti-rabbit serum. Antibodies purified from normal rabbit serum (NRS) were used as negative control.
(TIF)

S3 Fig Antibody-dependent respiratory burst assay. Human polymorphonuclear neutrophil granulocyte (PMN) responses towards purified PlasmoMix-specific rabbit immune IgG. Sample (R3_91, black squares) was analyzed using ADRB on coated antigens gAMA1 (A) and E3 (B) over a 60 min kinetic window with PNMs from one donor. Antibodies purified from normal rabbit serum (NRS) were used as negative control (red circles).
(TIF)

S1 Table Evaluation of transmission blocking activity of purified rabbit IgGs. Rabbit (R1, R2 and R3) were immunized with PlasmoMix and antibodies were purified from serum samples collected on days 35, 63 and 91. Polyclonal rabbit antibodies were used at a total IgG concentration of 1 mg/ml and based on the CFCA results, the F0-specific antibody concentration was calculated and listed. Antibodies purified from normal rabbit sera (NRS) was used as a negative control. Samples from day 63 and 91 were run in the same assay and refer to the same NRS, listed above R1_63. (a) We used a Mann-Whitney U test to analyze the median numbers of oocysts during infection with P. falciparum between groups of mosquitoes receiving either antibodies purified from NRS or purified from rabbits immunized with PlasmoMix. P-values below 0.05 were considered significant. (b) The prevalence of oocysts during the infection with P. falciparum between groups of mosquitoes receiving either antibodies purified from NRS or antibodies purified from rabbits immunized with PlasmoMix was analyzed using the Fisher’s exact test. P-values below 0.05 were considered significant.
(DOCX)

S2 Table Correlation of transmission blocking activity and F0-specific antibody concentration. Antibodies were purified from serum samples collected on day 91 after immunization with PlasmoMix and used at total IgG concentration of 1 mg/ml, 0.1 mg/ml and 0.01 mg/ml. The F0-specific antibody concentration was calculated based on the CFCA experiment. Antibodies purified from normal rabbit sera (NRS) was used as a negative control. Statistical analysis was performed as described in S1 Table.
(DOCX)
