# Supplementary figures and images for: Analysis of a Multi-component Multi-stage Malaria Vaccine Candidate—Tackling the Cocktail Challenge
Source: PLoS One. 2015 Jul 6;10(7):e0131456. doi: 10.1371/journal.pone.0131456 (PMC4492585; doi:10.1371/journal.pone.0131456)

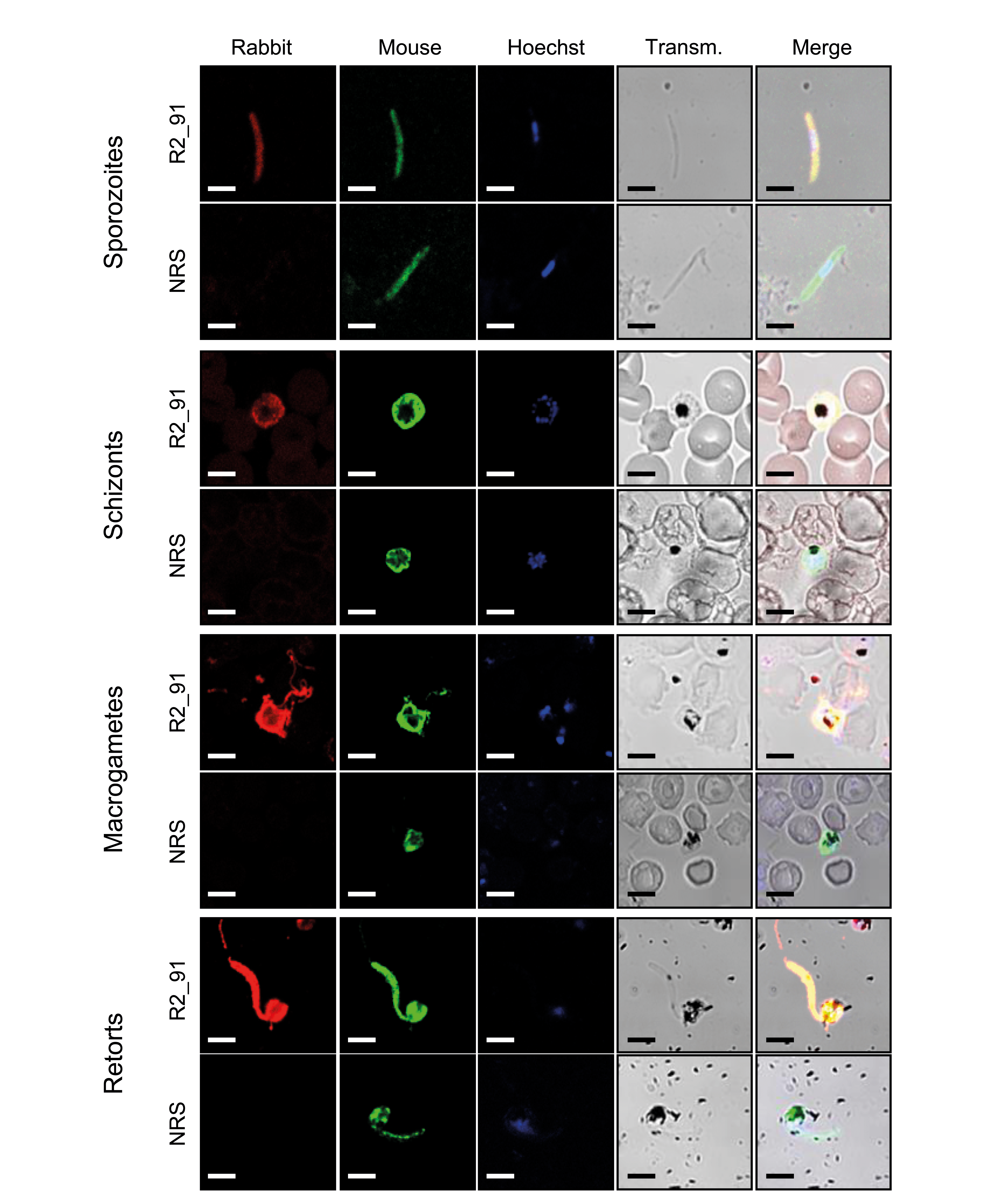

Supplement: S1 Fig — For IFAs, P. falciparum NF54 parasites in the sporozoite, schizont, macrogamete and retort stages were fixed with methanol on the surface of a slide. Exemplary shown is the detection with purified IgGs from rabbit R2 from the serum sample collected on day 91 after immunization with PlasmoMix. As positive controls, murine antisera against PfCSP (sporozoite), PfMSP1-19 (schizonts), Pfs25 (magrogametes) and Pfs28 (retorts) were used, respectively. Rabbit antibodies were visualized with anti-rabbit secondary antibody labeled with Alexa Fluor 594 (red) while visualization of murine antibodies was performed with secondary Alexa Fluor 488 labeled anti-murine antibody (green). Parasite nulcei were highlighted with Hoechst 33342 (blue). Transm: Transmission light. Bar: 5 μm. (TIF) [file pone.0131456.s001.tif]

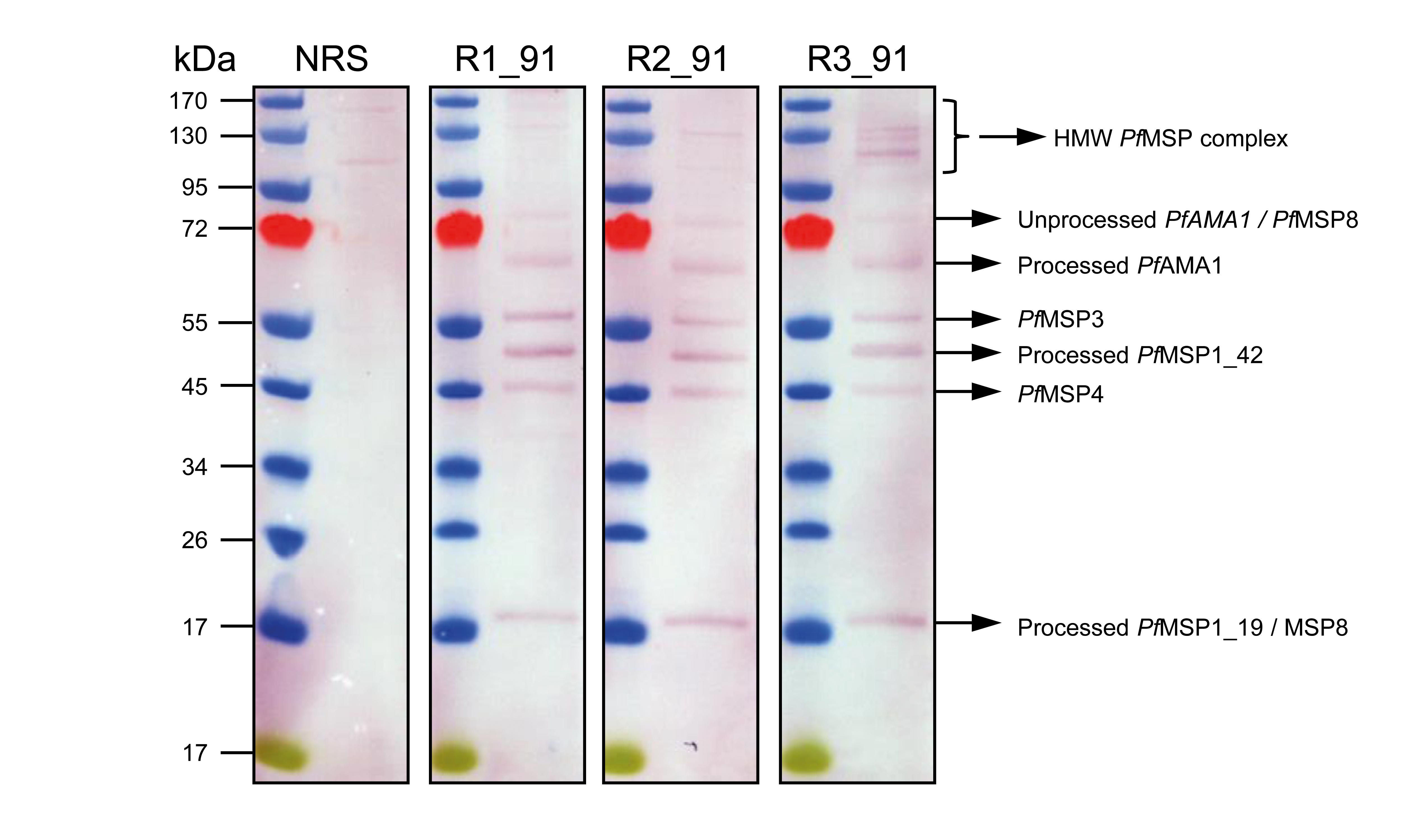

Supplement: S2 Fig — Purified rabbit immune IgG (R1, R2 and R3 from day 91) were used to detect native Plasmodium falciparum blood-stage antigens in immunoblot analysis with schizont lysate. 12 μl lysate (corresponding to 1.5x10^7^ schizonts) were separated under non-reducing conditions by SDS-PAGE. After immunoblot the membrane was blocked with skimmed milk and probed with rabbit immune IgG adjusted to 5 μg/ml PfAMA1-spacific IgG concentration. Bound rabbit antibodies were detected with an alkaline phosphatase goat anti-rabbit serum. Antibodies purified from normal rabbit serum (NRS) were used as negative control. (TIF) [file pone.0131456.s002.tif]

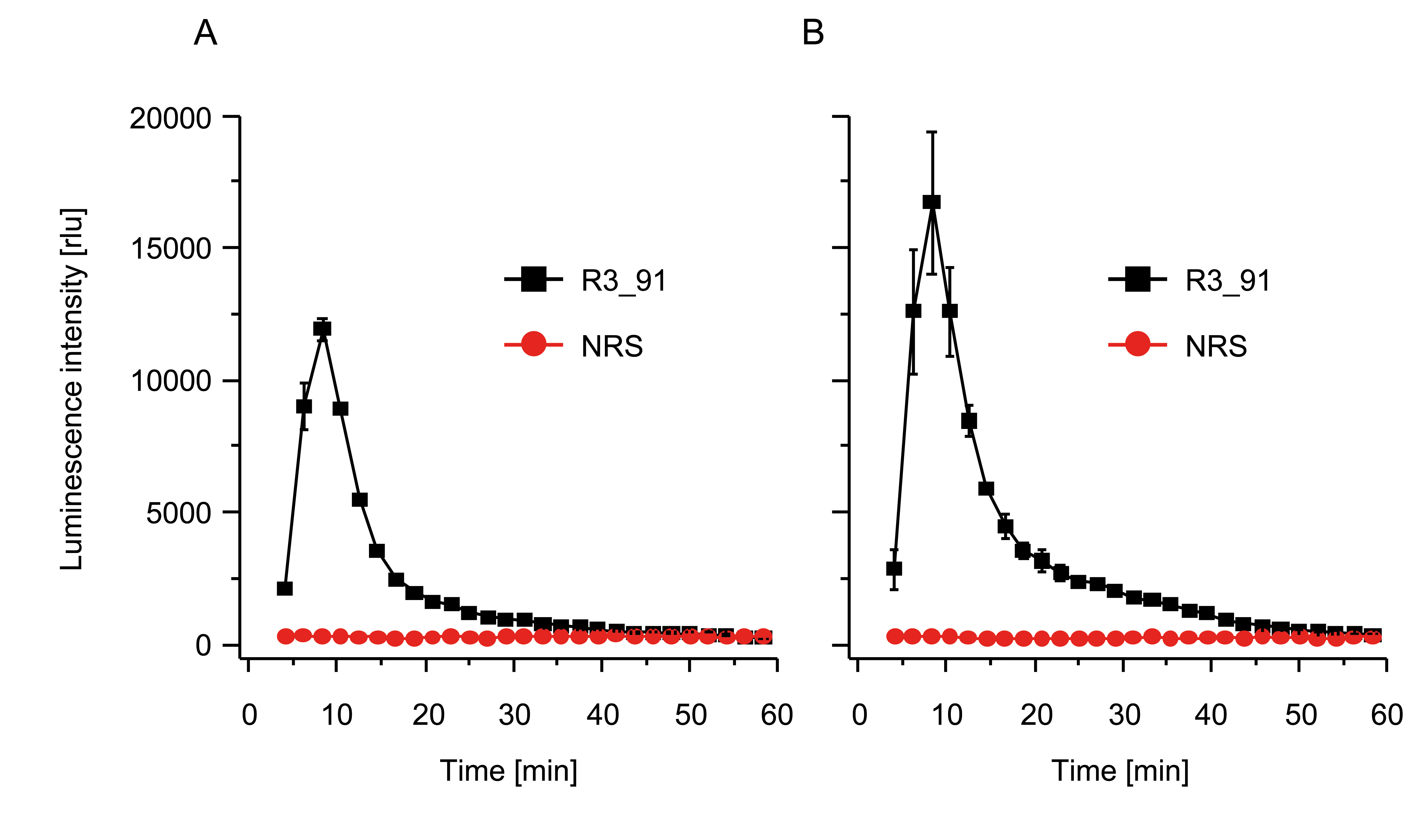

Supplement: S3 Fig — Human polymorphonuclear neutrophil granulocyte (PMN) responses towards purified PlasmoMix-specific rabbit immune IgG. Sample (R3_91, black squares) was analyzed using ADRB on coated antigens gAMA1 (A) and E3 (B) over a 60 min kinetic window with PNMs from one donor. Antibodies purified from normal rabbit serum (NRS) were used as negative control (red circles). (TIF) [file pone.0131456.s003.tif]
