# Supplementary material for: Analysis of a Multi-component Multi-stage Malaria Vaccine Candidate—Tackling the Cocktail Challenge
Source: PLoS One. 2015 Jul 6;10(7):e0131456. doi: 10.1371/journal.pone.0131456 (PMC4492585; doi:10.1371/journal.pone.0131456)
Supplement: S1 Table — Rabbit (R1, R2 and R3) were immunized with PlasmoMix and antibodies were purified from serum samples collected on days 35, 63 and 91. Polyclonal rabbit antibodies were used at a total IgG concentration of 1 mg/ml and based on the CFCA results, the F0-specific antibody concentration was calculated and listed. Antibodies purified from normal rabbit sera (NRS) was used as a negative control. Samples from day 63 and 91 were run in the same assay and refer to the same NRS, listed above R1_63. (a) We used a Mann-Whitney U test to analyze the median numbers of oocysts during infection with P. falciparum between groups of mosquitoes receiving either antibodies purified from NRS or purified from rabbits immunized with PlasmoMix. P-values below 0.05 were considered significant. (b) The prevalence of oocysts during the infection with P. falciparum between groups of mosquitoes receiving either antibodies purified from NRS or antibodies purified from rabbits immunized with PlasmoMix was analyzed using the Fisher’s exact test. P-values below 0.05 were considered significant. (DOCX) [file pone.0131456.s004.docx]

S1 Table

| **Sample** | **Concentration**  **Total IgG [mg/ml]** | **Concentration**  **F0-specifc IgG**  **[µg/ml]** | **Mean number of oocysts (range)** | **p-values ^a^** | **Mosquitoes infected/dissected** | **Inhibition of transmission [%]** | **p-values ^b^** |
| --- | --- | --- | --- | --- | --- | --- | --- |
| NRS | 1 | 0 | 3 (0-7) | - | 12/20 | - | - |
| R1_35 | 1 | 11.8 | 0 | 0.00142 | 0/19 | 100 | 0.000045 |
| R2_35 | 1 | 12.6 | 1 (0-1) | 0.00214 | 1/20 | 95 | 0.000432 |
| R3_35 | 1 | 22.5 | 0 | 0.00124 | 0/20 | 100 | 0.000045 |
| NRS | 1 | 0 | 3 (0-10) | - | 14/20 | - | - |
| R1_63 | 1 | 15.6 | 0 | 0.00016 | 0/20 | 100 | 0.000003 |
| R2_63 | 1 | 21.1 | 0 | 0.00016 | 0/20 | 100 | 0.000003 |
| R3_63 | 1 | 16.2 | 0 | 0.00016 | 0/20 | 100 | 0.000003 |
| R1_91 | 1 | 10.2 | 1 (0-1) | 0.00026 | 1/20 | 95 | 0.000039 |
| R2_91 | 1 | 17.5 | 0 | 0.00016 | 0/20 | 100 | 0.000003 |
| R3_91 | 1 | 15.6 | 0 | 0.00016 | 0/20 | 100 | 0.000003 |
