# Supplementary material for: Analysis of a Multi-component Multi-stage Malaria Vaccine Candidate—Tackling the Cocktail Challenge
Source: PLoS One. 2015 Jul 6;10(7):e0131456. doi: 10.1371/journal.pone.0131456 (PMC4492585; doi:10.1371/journal.pone.0131456)
Supplement: S2 Table — Antibodies were purified from serum samples collected on day 91 after immunization with PlasmoMix and used at total IgG concentration of 1 mg/ml, 0.1 mg/ml and 0.01 mg/ml. The F0-specific antibody concentration was calculated based on the CFCA experiment. Antibodies purified from normal rabbit sera (NRS) was used as a negative control. Statistical analysis was performed as described in S1 Table. (DOCX) [file pone.0131456.s005.docx]

S2 Table

| **Sample** | **Concentration**  **Total IgG [mg/ml]** | **Concentration**  **F0-specifc IgG**  **[µg/ml]** | **Mean number of oocysts (range)** | **p-values ^a^** | **Mosquitoes infected/dissected** | **Inhibition of transmission**  **[%]** | **p-values ^b^** |
| --- | --- | --- | --- | --- | --- | --- | --- |
| NRS | 1 | 0 | 3 (0-10) | - | 14/20 | - | - |
| R1_91 | 1 | 10.2 | 1 (0-1) | 0.00026 | 1/20 | 95 | 0.000039 |
| R2_91 | 1 | 17.5 | 0 | 0.00016 | 0/20 | 100 | 0.000003 |
| R3_91 | 1 | 15.6 | 0 | 0.00016 | 0/20 | 100 | 0.000003 |
| NRS | 0.1 | 0 | 19 (6-37) | - | 20/20 | - | - |
| R1_91 | 0.1 | 1.02 | 6 (3-12) | 0 | 20/20 | 0 | 1 |
| R2_91 | 0.1 | 1.75 | 2 (0-3) | 0 | 11/20 | 45 | 0.001229 |
| R3_91 | 0.1 | 1.56 | 2 (0-3) | 0 | 12/20 | 40 | 0.003276 |
| NRS | 0.01 | 0 | 23 (7-47) | - | 20/20 | - | - |
| R1_91 | 0.01 | 0.102 | 21 (2-51) | 0.69654 | 20/20 | 0 | 1 |
| R2_91 | 0.01 | 0.175 | 22 (4-47) | 0.96012 | 20/20 | 0 | 1 |
| R3_91 | 0.01 | 0.156 | 19 (7-45) | 0.32218 | 20/20 | 0 | 1 |
